# Siglec-9 acts as an immune checkpoint marker on MDSCs in brucella infection

**DOI:** 10.3389/fcimb.2025.1652116

**Published:** 2025-10-29

**Authors:** Huidong Shi, Xinxin Qi, Kaiyu Shang, Tingting Tian, Jianbing Ding, Mingzhe Li, Ruixue Xu, Fuling Pu, Junyu Kuang, Yuejie Zhu, Fengbo Zhang

**Affiliations:** ^1^ State Key Laboratory of Pathogenesis, Prevention and Treatment of High Incidence Diseases in Central Asia, The First Affiliated Hospital of Xinjiang Medical University, Urumqi, China; ^2^ Department of Clinical Laboratory, The First Affiliated Hospital of Xinjiang Medical University, Urumqi, China; ^3^ School of Public Health, Xinjiang Medical University, Urumqi, China; ^4^ Department of Medical Laboratory Technology, School of Medicine, Xinjiang Medical University, Urumqi, China; ^5^ Reproductive Medicine Center, The First Affiliated Hospital of Xinjiang Medical University, Urumqi, China

**Keywords:** Brucellosis, MDSCs, PD-1, Tim-3, Siglec-9, Cytokines

## Abstract

**Background:**

Brucellosis is a zoonotic disease that is widely prevalent in the Xinjiang region of China. Once it progresses to a chronic stage, it can lead to significant complications. Immune checkpoints markers on Myeloid-derived suppressor cells (MDSCs) may lead to the chronic stage of the disease. This study analyzed the changes in MDSCs, immune checkpoints markers and cytokines in the patients with acute and chronic Brucella infections and after antibiotic treatment, to explore their roles and provide new ideas for future clinical treatment.

**Methods:**

A total of 37 patients with acute brucellosis infection (ABI) and 46 patients with chronic brucellosis infection (CBI) and 43 healthy controls (HC) subjects were enrolled. Flow cytometry was used to detect the expression of MDSCs, Siglec-9^+^MDSCs, PD-1^+^MDSCs and Tim-3^+^MDSCs before and after antibiotic treatment. In addition, ELISA was used to measure the levels of cytokines and the changes in IL-6 and Arg-1 of them were assessed again after antibiotic treatment.

**Results:**

Our study found that the levels of MDSCs in the patients significantly increased, with CBI patients exhibiting higher levels than ABI patients. The cytokines showed varying degrees of elevation. Furthermore, after antibiotic treatment, the levels of MDSCs, Siglec-9^+^ MDSCs, PD-1^+^ MDSCs and Tim-3^+^ MDSCs in effective treatment patients significantly decreased. In contrast, the levels of MDSCs in ineffective treatment patients increased, while there were no significant differences in PD-1^+^ MDSCs and Tim-3^+^ MDSCs levels compared to before treatment. Notably, the levels of Siglec-9^+^MDSCs in ineffective treatment showed a significant increase. In the ineffective treatment patients, the serum levels of Arg-1 and IL-6 both increased compared to before treatment. Correlation analysis revealed a positive correlation in ineffective treatment patients between serum Arg-1 levels and MDSCs, as well as Siglec-9^+^ MDSCs levels, while no correlation was observed between IL-6 levels and immune cell parameters.

**Conclusion:**

MDSCs are increased in both ABI and CBI. Siglec-9 acts as an immune checkpoint on MDSCs in patients with ineffective treatment responses. Therefore, Siglec-9 represents a potential prognostic marker for Brucella infection. Ongoing research on prognostic markers of brucellosis is promising, and further clinical studies are warranted to validate these findings.

## 1 Introduction

Brucellosis is a zoonotic disease that poses significant threats to human biosafety and economic development worldwide. The World Health Organization currently classifies brucellosis as one of the most easily overlooked zoonotic diseases globally. The Xinjiang region of China is the main epidemic area for brucellosis, with sheep and goats being the main sources of infection (Wang et al., 2024). Human brucellosis can affect multiple organs and even develop systemic systems. After infection, patients typically enter the acute phase of the disease, where they often exhibit non-specific symptoms such as fever, sweating, chills, and hearing impairment. If patients do not receive timely treatment, their condition may progress to the chronic phase, presenting specific symptoms such as arthritis, hepatomegaly, and splenomegaly (Qiangsheng et al., 2023; Zheng et al., 2018). Meanwhile, the lack of effective treatment methods and vaccines for patients may lead to treatment failures, exacerbating the severity of the disease. Recently, Hou et al. discovered that levels of Myeloid-derived suppressor cells (MDSCs), IL-6, IL-10, and Arg-1 in the patients with CBI were significantly elevated, which may be related to the immunosuppressive state of patients with CBI (Hou et al., 2024a).

MDSCs originate from myeloid progenitor cells and represent a highly suppressive cell population. In humans, MDSCs are primarily found in the blood, tumors, and various organs. The phenotypic characteristics of MDSCs described as CD45^+^HLA-DR^-/lo^ CD33^+^CD11b^+^ (He et al., 2025). Increasing evidence suggests that the accumulation of MDSCs is associated with chronic inflammation and plays an immunosuppressive role in infectious diseases (Li et al., 2020; Zhu and Cao, 2025). As an immunosuppressive cell, MDSCs mainly function by overexpressing immune checkpoint markers and secreting Arg-1 and iNOS (Barry et al., 2023; Cao et al., 2022). Recent studies have emphasized the impact of immune checkpoints markers on the function of MDSCs in various disease contexts, including PD-1, Tim-3, and Siglec-9. However, the functional performance of immune checkpoints markers on MDSCs has not yet been described in brucellosis.

Siglecs have emerged as a novel immune checkpoint. The human Siglecs family consists of 14 members, which can be divided into two groups: the conserved Siglecs (1, 2, 4, 15) and the CD33-related Siglecs (3, 5-11, 14, 16). The conserved Siglecs exhibit high sequence similarity across species, while the CD33-related Siglecs demonstrate significant interspecies sequence variability (van Houtum et al., 2021; Santegoets et al., 2019). The research has found that Siglec-9 acts as an inhibitory receptor that regulates the activation of mast cells (Miralda et al., 2023). In the mouse model of sepsis, the Siglec-F^+^ neutrophils that are highly expressed can inhibit T lymphocytes (Liao et al., 2024). Studies also have shown that MDSCs in the TME express high levels of the sialic acid receptor Siglec-E, which significantly enhances the immunosuppressive function of MDSCs (Wieboldt et al., 2024). Therefore, targeting immune checkpoints markers may hold great promise for improving patient immunosuppression.

There are few reports on the interaction between MDSCs and immune checkpoints markers in infectious diseases, particularly in chronic infections. This study identifies CD33^+^CD11b^+^ as a marker for MDSCs and analyzes of patients with acute brucellosis infection (ABI) , chronic brucellosis infection (CBI) and healthy controls(HC) subjects. Additionally, the expression of immune checkpoint markers (Siglec-9, Tim-3, and PD-1) on MDSCs also examined. Subsequently, the concentrations of IL-10, IL-6, TGF-β, IFN-γ, Arg-1, and iNOS were measured using ELISA in patients with ABI, CBI and HC subjects. Finally, after antibiotic treatment, the expression of MDSCs and immune checkpoints markers was re-evaluated. The findings suggest that the novel immune checkpoint marker Siglec-9 may serve as a prognostic target for patients suffering from brucellosis.

## 2 Materials and methods

### 2.1 Study subjects

From January 2024 to February 2025, we collected data from 37 ABI patients with a disease duration of less than 3 months and 46 CBI patients with a disease duration of more than 6 months at the First Affiliated Hospital of Xinjiang Medical University. Simultaneously, 43 HC subjects who underwent examinations at the same hospital during this period were enrolled as controls. All participants signed informed consent forms, and the study was approved by the Ethics Committee of the First Affiliated Hospital of Xinjiang Medical University (Ethics Approval No: K202409-16).

### 2.2 Inclusion and exclusion criteria

Inclusion criteria: 1. Diagnosis of confirmed cases according to the ‘Diagnosis of Brucellosis’ (WS 269—2019), with positive serological responses; 2. Cases with a disease duration of less than 3 months are classified as acute, while those exceeding 6 months are classified as chronic; 3. The control group consists of HC undergoing health check-ups; 4. Complete data is required. Exclusion criteria: 1. Patients with infectious diseases such as typhoid fever, paratyphoid fever, and tuberculosis; 2. Patients with underlying diseases and tumors; 3. Individuals who have used immunomodulatory or immunosuppressive drugs for a prolonged period or within the last 3 months; 4. Patients who do not consent to participate in this study.

### 2.3 Flow cytometry

Peripheral blood mononuclear cells (PBMCS) were prepared from fresh blood samples by Ficoll density sedimentation. The isolated PBMC was incubated with fluorescently labeled monoclonal antibodies at 4 °C for 30 minutes. MDSCs were labeled by CD45, HLA-DR, CD33, CD11b. Meanwhile, the expression of PD-1, Tim-3 and Siglec-9 on MDSCs was evaluated. After surface staining, the cells were washed twice with flow cytometry buffer and then put on the machine. Data were obtained using the BD FACSLyric™ flow cytometer. After obtaining the data, the FlowJo software is used to analyze it. MDSCs are defined as CD33^+^CD11b^+^ cells. The information on the use of antibodies is shown in **Table 1**.

**Table 1:** Antibodies for flow cytometry.

Antibodies	Clone	Source	Catalog number
APC/Cyanine7 anti-human CD45 Antibody	HI30	Biolegend	304014
Brilliant Violet 510™ anti-human HLA-DR Antibody	L243	Biolegend	307646
APC Mouse Anti-Human CD33	WM53	BD Biosciences	551378
PerCP anti-mouse/human CD11b Antibody	M1/70	Biolegend	101230
Brilliant Violet 421™ anti-human CD14 Antibody	HCD14	Biolegend	325628
Brilliant Violet 605™ anti-human CD15 (SSEA-1) Antibody	W6D3	Biolegend	323032
BV605 Mouse Anti-Human CD279 (PD-1)	EH12.1	BD Biosciences	563245
CD366 (TIM3) Monoclonal Antibody	F38-2E2	eBioscience	25-3109-42
PE anti-human Siglec-9 Antibody	K8	Biolegend	351504

### 2.4 Enzyme linked immunosorbent assay

Serum samples from all subjects were stored at -80 °C. The concentrations of IL-10, IL-6, TGF-β, IFN-γ, Arg-1, and iNOS were measured using ELISA kits, and the optical density (OD) values of the sample reactions were measured at 450 nm using a microplate reader. The information on the use of ELISA kits is shown in **Table 2**.

**Table 2:** Cytokines for enzyme- linked immunosorbent assay.

Cytokines	Source	Catalog number
IL-6	JONLNBIO	JL14113
IL-10	JONLNBIO	JL19246
TGF-β	JONLNBIO	JL20082
IFN-γ	JONLNBIO	JL12152
Arg-1	JONLNBIO	JL19534
iNOS	JONLNBIO	JL20025

### 2.5 Statistic analysis

The Shapiro-Wilk test was used to determine whether the data followed a normal distribution. The results are expressed as mean ± standard deviation. When comparing between the two groups, the t-test was used for normally distributed variables, the Wilcoxon rank-sum test was used for non-normally distributed variables, and one-way analysis of variance(ANOVA) was used for the differences among multiple groups of data. Pearson correlation analysis was adopted. Statistical analysis was performed using GraphPad Prism 9.5.0. P value <0.05 was considered significant.

## 3 Result

### 3.1 Demographic and clinical characteristics

This study included a total of 37 patients with ABI, aged between 20 and 65 years, with a mean age of 42.16 ± 9.31 years, comprising 31 males and 6 females. The CBI group consisted of 46 patients, aged 21 to 65 years, with a mean age of 41.39 ± 10.26 years, including 40 males and 6 females. The HC group included 43 individuals, aged 19 to 63 years, with a mean age of 42.35 ± 8.79 years, consisting of 36 males and 7 females. There were no statistically significant differences in gender, age, and ethnicity among the three groups. The demographic and clinical characteristics of the ABI, CBI and HC are presented in **Table 3** and **Table 4**.

**Table 3:** Demographic characteristics of ABI, CBI and HC during sample collection.

Variable	HC (%), n=43	ABI (%), n=37	CBI (%), n=46
Sex			
Male	36 (83.7)	31 (83.8)	40 (87.0)
Female	7 (16.3)	6 (16.2)	6 (13.0)
Age (years)			
19-35	13 (30.2)	11 (29.7)	14 (30.4)
36-50	21 (48.8)	18 (48.6)	23 (50.0)
51-65	9 (20.9)	8 (21.6)	9 (19.6)
Ethnicity			
Han	10 (23.3)	7 (18.9)	11 (23.9)
Uyghur	20 (46.5)	18 (48.6)	21 (45.7)
Hui	6 (14.0)	4 (10.8)	7 (15.2)
Kazakh	4 (9.3)	3 (8.1)	4 (8.7)
Mongolian	2 (4.7)	3 (8.1)	2 (4.3)
Kyrgyz	1 (2.3)	2 (5.4)	1 (2.2)
Area			
Urban	15 (34.9)	5 (13.5)	10 (21.7)
Rural	28 (65.1)	32 (86.5)	36 (78.3)

**Table 4:** The clinical and laboratory examination results of ABI and CBI and HC groups during sample collection.

Clinical symptoms	HC (%) n=43	ABI (%) n=37	CBI (%) n=46
Fever (>37.3°C)	-	35 (94.6)	40 (87.0)
Fatigue	-	34 (91.9)	46 (100.0)
Hepatomegaly or splenomegaly	-	15 (40.5)	35 (76.1)
Myalgia or arthralgia	-	20 (54.1)	30 (65.2)
Lymphadenopathy	-	28 (75.7)	37 (80.4)
Orchitis	-	16 (43.2)	26 (56.5)
WBC> (3.97-9.15)x10^9^/L	-	15 (40.5)	23 (50.0)
ALT>50 (U/L)	-	10 (27.0)	18 (39.1)
AST>59 (U/L)	-	8 (21.6)	13 (28.3)
CRP>8 (mg/L)	-	29 (78.4)	39 (84.8)
ESR^a^	-	30 (81.8)	37 (80.4)

a ESR: Male > 15 mm/h, Female > 20 mm/h.

### 3.2 Upregulation of immune checkpoints markers on MDSCs in patients with brucellosis

**Figure 1:**
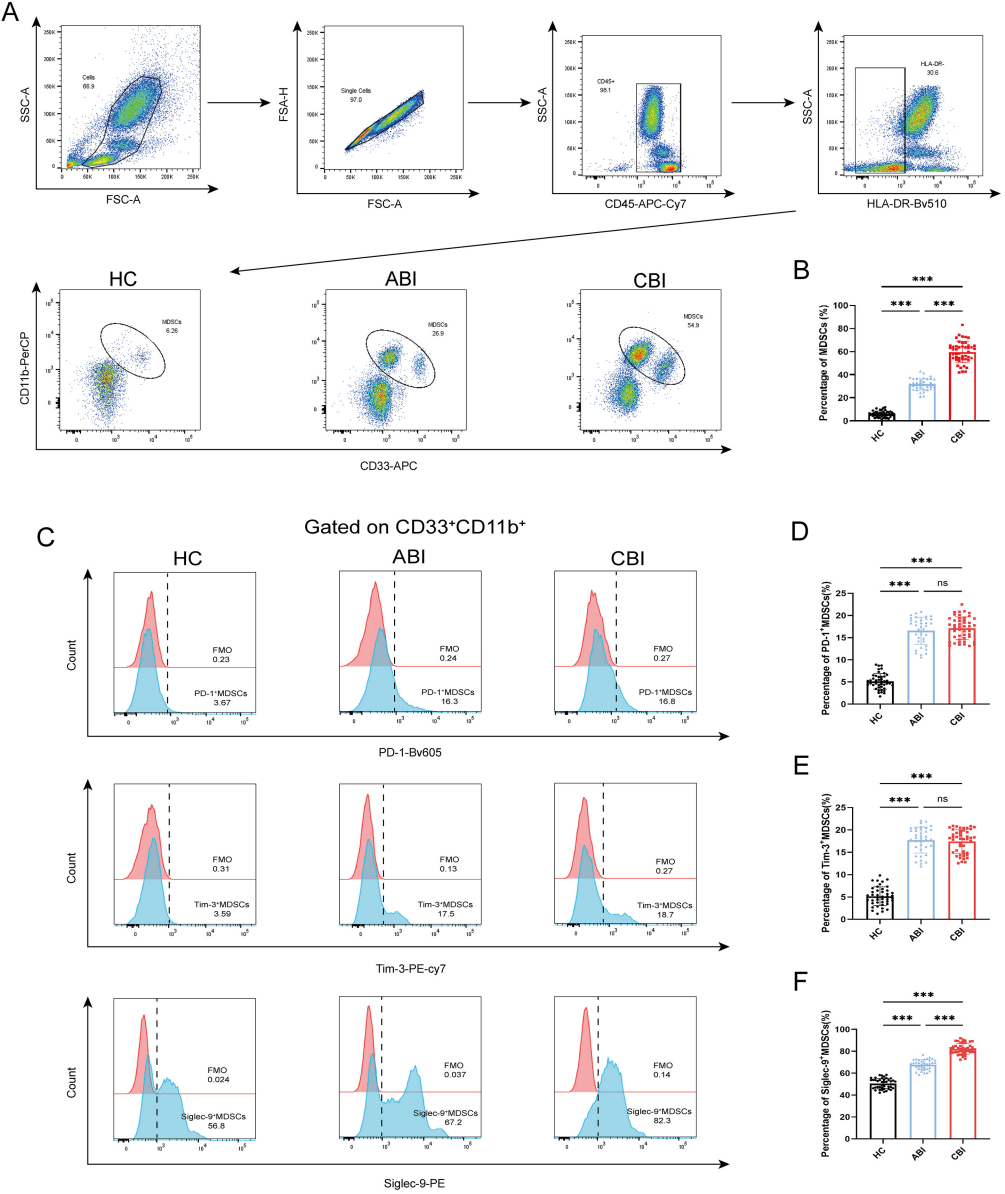
Percentage of MDSCs, PD-1^+^ MDSCs, Tim-3^+^ MDSCs and Siglec-9^+^ MDSCs in the HC, ABI and CBI group. **(A)** Flow cytometry gating strategies for MDSCs. **(B)** Comparison of the percentage of MDSCs between 43 HC subjects and 37 ABI patients and 46 CBI patients. **(C)** Flow cytometry gating strategies for PD-1^+^ MDSCs, Tim-3^+^ MDSCs and Siglec-9^+^ MDSCs. **(D)** Comparison of the percentage of PD-1^+^ MDSCs between 43 HC subjects and 37 ABI patients and 46 CBI patients. **(E)** Comparison of the percentage of Tim-3^+^ MDSCs between 43 HC subjects and 37 ABI patients and 46 CBI patients. **(F)** Comparison of the percentage of Siglec-9^+^ MDSCs between 43 HC subjects and 37 ABI patients and 46 CBI patients. ^***^P<0.001, ns, no significant difference.

MDSCs are defined as CD33^+^CD11b^+^ cells by flow cytometry. We compared the percenrage of MDSCs, Siglec-9^+^ MDSCs, Tim-3^+^ MDSCs, PD-1^+^ MDSCs among 43 HC subjects, 37 ABI patients and 46 CBI patients (**Figures 1A, C**). The results showed that the percenrage of MDSCs in both ABI patients and CBI patients was higher than that in HC subjects and CBI patients is higher than that in ABI patients. (**Figure 1B**, P<0.001). Furthermore, the percenrage of PD-1^+^ MDSCs and Tim-3^+^ MDSCs in both ABI patients and CBI patients was higher than that in HC subjects (**Figures 1D, E**, P<0.001), but there was no difference between ABI patients and CBI patients (**Figures 1D, E**, ns). However, the percenrage of Siglec-9^+^ MDSCs in CBI patients was higher than ABI patients (**Figure 1F**, P<0.001).

### 3.3 Increased serum IL-10, IL-6, TGF-β, IFN-γ, Arg-1 and iNOS levels in patients with brucellosis

**Figure 2:**
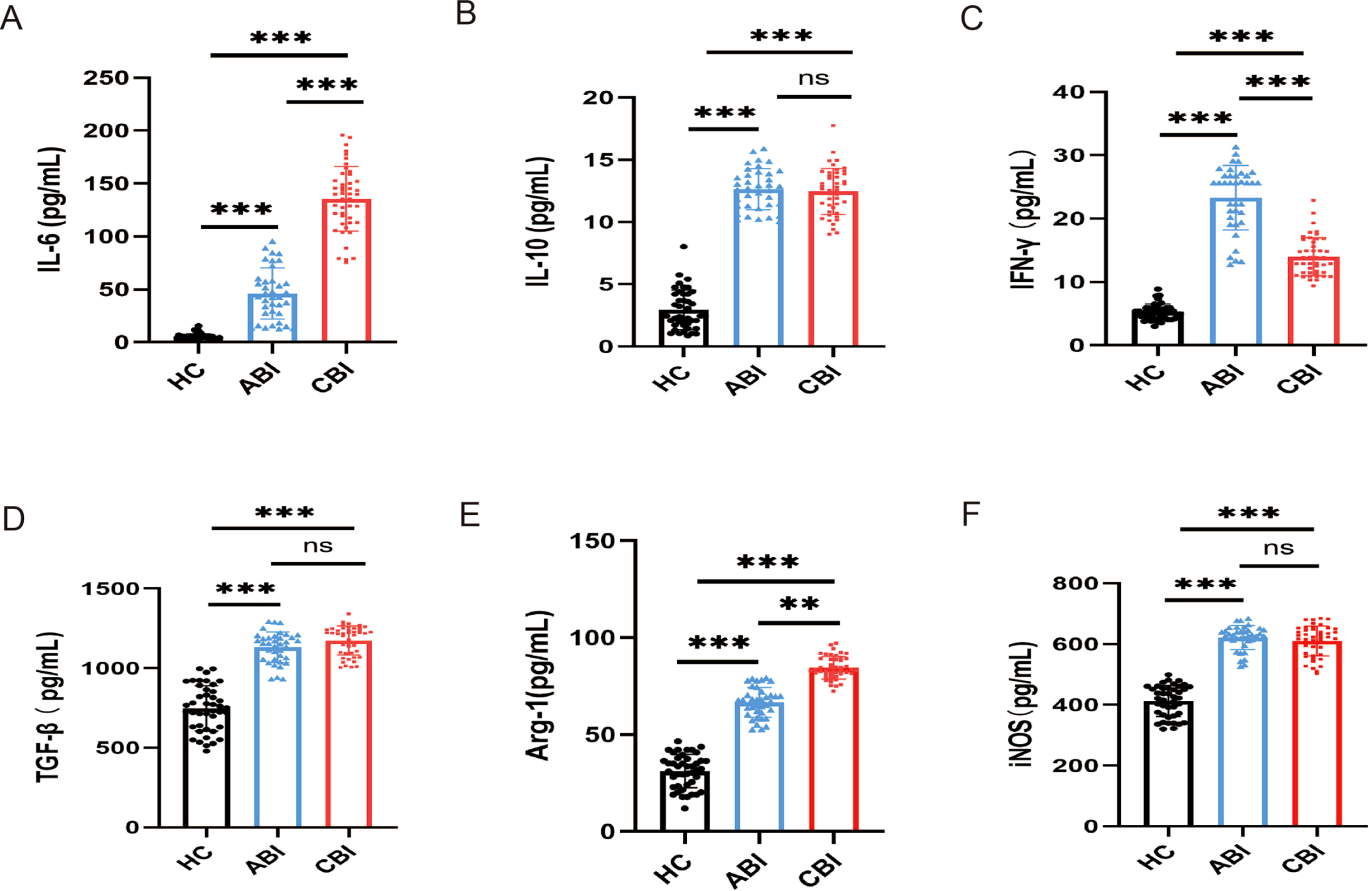
ELISA shows the concentrations of inflammation-related cytokines in the serum of ABI group (n=37), CBI group (n=46), HC group (n=43). **(A)** IL-6 **(B)** IL-10 **(C)** IFN-γ **(D)** TGF-β **(E)** Arg-1 **(F)** iNOS. ^**^P<0.01, ^***^P<0.001, ns, no significant difference.

Using the ELISA to detecte the levels of inflammation-related cytokines (IL-10, IL-6, TGF-β, IFN-γ, Arg-1and iNOS) in the serum of groups with ABI and CBI and HC. The results indicated that the levels of IL-6 and Arg-1 were elevated in the serum of brucellosis infected patients, with CBI group exhibiting higher levels than ABI group (**Figures 2A, E**, P<0.01,P<0.001). IFN-γ was highest during the acute phase and showed a decline in the chronic phase (**Figure 2C**, P<0.001). The levels of IL-10, TGF-β, and iNOS in ABI and CBI groups were higher than those in HC group, but no significant differences were observed between ABI and CBI group (**Figures 2B, D, F**, P<0.001).

### 3.4 Observation of MDSCs, immune checkpoints markers and cytokine changes in patients after antibiotic treatment

**Figure 3:**
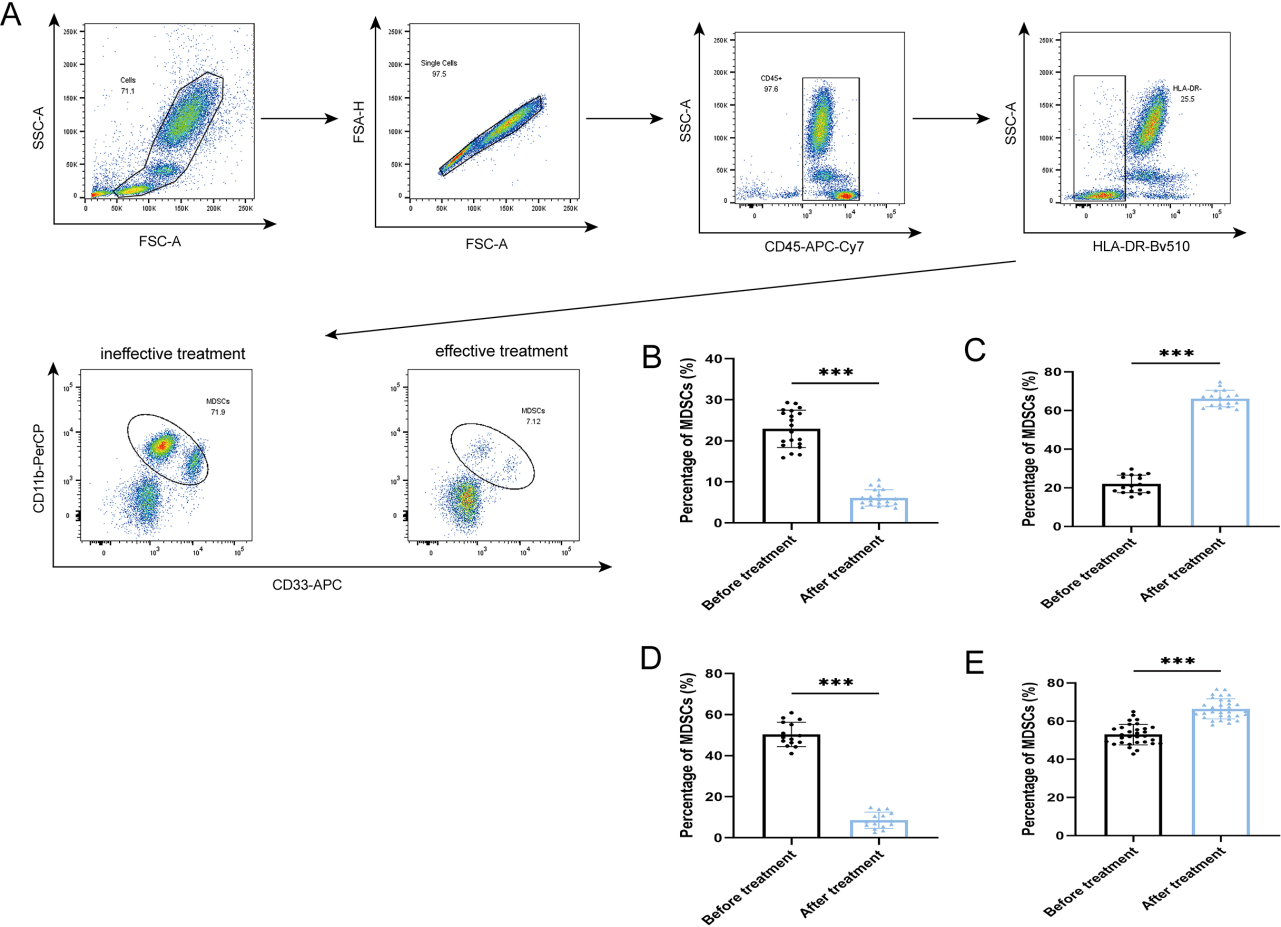
Percentage of MDSCs in the effective treatment and ineffective treatment group **(A)** Flow cytometry gating strategies for MDSCs. **(B)** Comparison of the percentage of MDSCs of 20 ABI patients with effective antibiotic treatment. **(C)** Comparison of the percentage of MDSCs of 17 ABI patients with ineffective antibiotic treatment. **(D)** Comparison of the percentage of MDSCs of 15 CBI patients with effective antibiotic treatment. **(E)** Comparison of the percentage of MDSCs of 31 CBI patients with ineffective antibiotic treatment. ^***^P<0.001.

**Figure 4:**
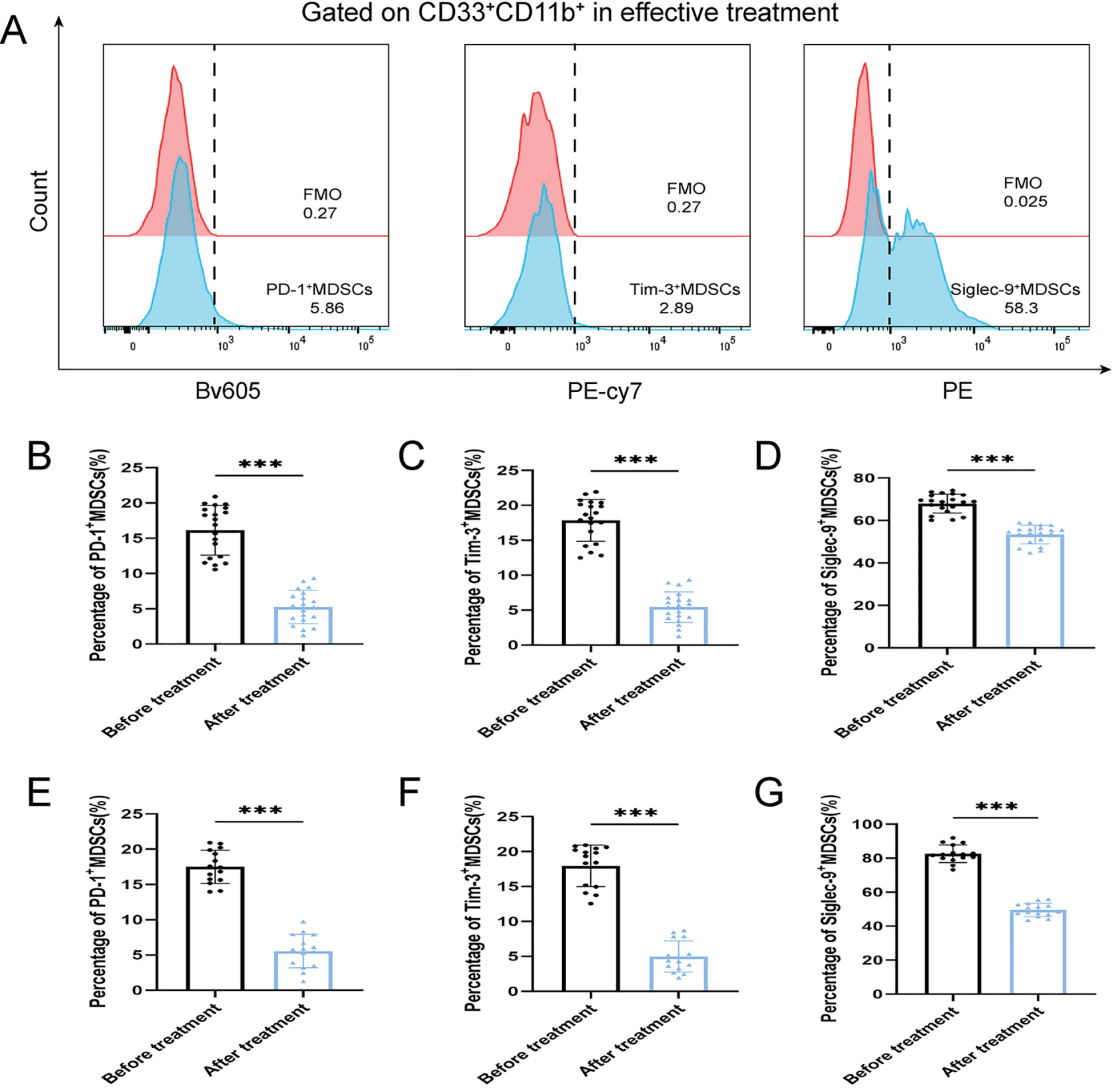
Percentage of PD-1^+^ MDSCs, Tim-3^+^ MDSCs and Siglec-9^+^ MDSCs of ABI and CBI patients after effective antibiotic treatment. **(A)** Flow cytometry gating strategies for PD-1^+^ MDSCs, Tim-3^+^ MDSCs and Siglec-9^+^ MDSCs. **(B)** Comparison of the percentage of PD-1^+^ MDSCs of 20 ABI patients after effective antibiotic treatment. **(C)** Comparison of the percentage of Tim-3^+^ MDSCs of 20 ABI patients after effective antibiotic treatment. **(D)** Comparison of the percentage of Siglec-9^+^ MDSCs of 20 ABI patients after effective antibiotic treatment. **(E)** Comparison of the percentage of PD-1^+^ MDSCs of 15 CBI patients after effective antibiotic treatment. **(F)** Comparison of the percentage of Tim-3^+^ MDSCs of 15 CBI patients after effective antibiotic treatment. **(G)** Comparison of the percentage of Siglec-9^+^ MDSCs of 15 CBI patients after effective antibiotic treatment. ^***^P < 0.001.

**Figure 5:**
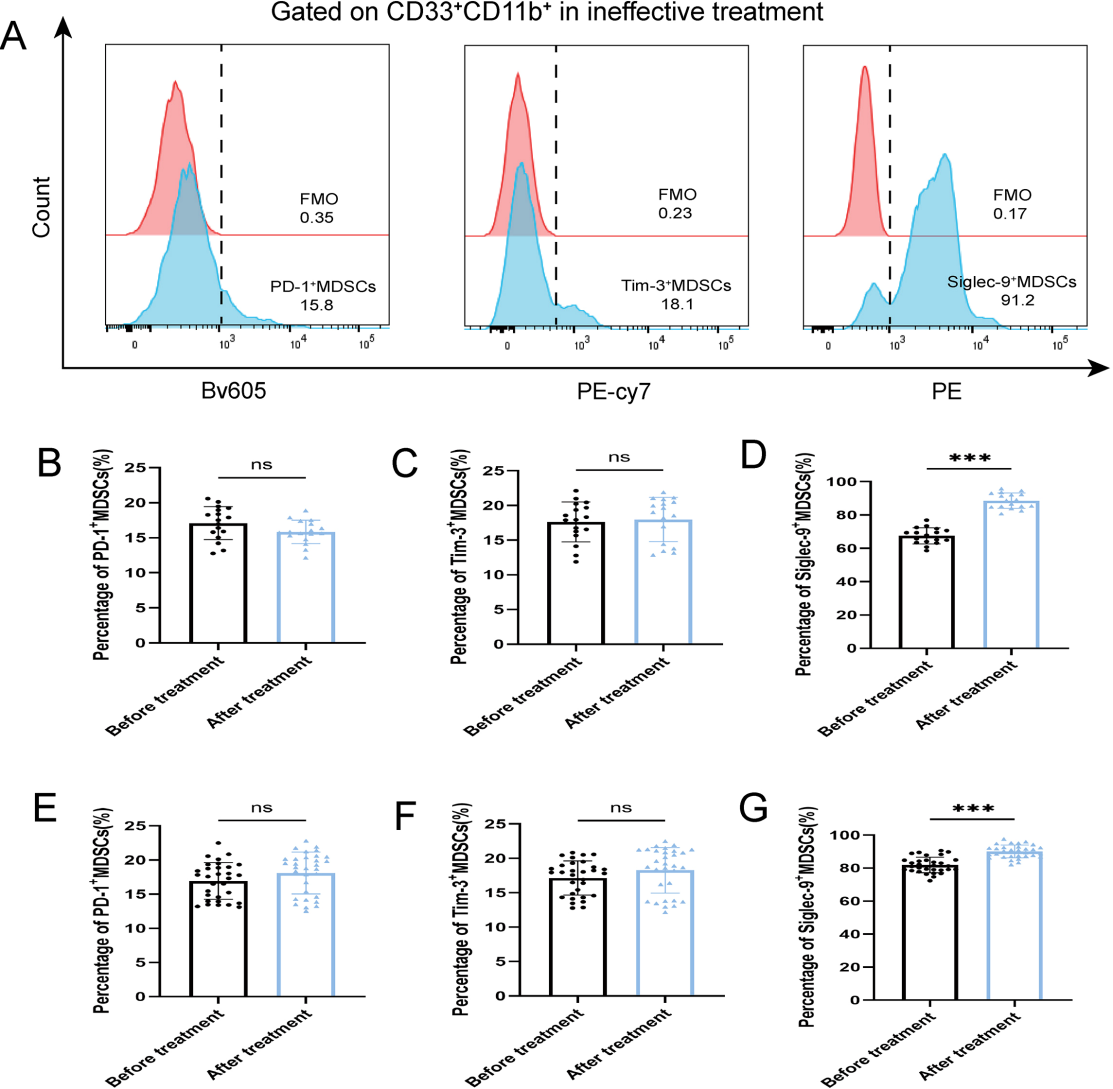
Percentage of PD-1^+^ MDSCs, Tim-3^+^ MDSCs and Siglec-9^+^ MDSCs of ABI and CBI patients after ineffective antibiotic treatment. **(A)** Flow cytometry gating strategies for PD-1^+^ MDSCs, Tim-3^+^ MDSCs and Siglec-9^+^ MDSCs. **(B)** Comparison of the percentage of PD-1^+^ MDSCs of 17 ABI patients after ineffective antibiotic treatment. **(C)** Comparison of the percentage of Tim-3^+^ MDSCs of 17 ABI patients after ineffective antibiotic treatment. **(D)** Comparison of the percentage of Siglec-9^+^ MDSCs of 17 ABI patients after ineffective antibiotic treatment. **(E)** Comparison of the percentage of PD-1^+^ MDSCs of 31 CBI patients after ineffective antibiotic treatment. **(F)** Comparison of the percentage of Tim-3^+^ MDSCs of 31 CBI patients after ineffective antibiotic treatment. **(G)** Comparison of the percentage of Siglec-9^+^ MDSCs of 31 CBI patients after ineffective antibiotic treatment. ^***^P < 0.001, ns, no significant difference.

**Figure 6:**
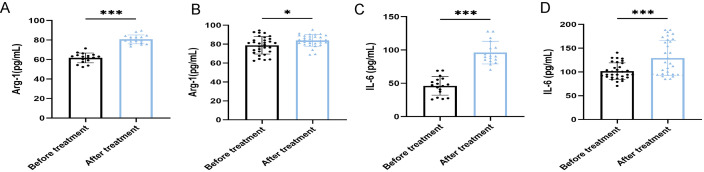
ELISA shows the serum concentrations of Arg-1 and IL-6 in patients with ineffective antibiotic treatment (n=48). **(A)** Comparison of the concentrations of Arg-1 of 17 ABI after ineffective antibiotic treatment. **(B)** Comparison of the concentrations of Arg-1 of 31 CBI after ineffective antibiotic treatment. **(C)** Comparison of the concentrations of IL-6 of 17 ABI after ineffective antibiotic treatment. **(D)** Comparison of the concentrations of IL-6 of 31 CBI after ineffective antibiotic treatment. *P<0.05, ***P<0.001.

After antibiotic treatment of ABI and CBI groups, it was found that among 37 patients with acute antibiotic treatment, 20 patients showed clinical improvement, while 17 patients experienced deterioration, progressing to a chronic state. Among the 46 patients with chronic antibiotic treatment, only 15 patients showed clinical improvement, while 31 patients still had chronic symptoms. Flow cytometry was used again to analyze the expression of MDSCs and different immune checkpoint markers in the patients (**Figures 3**-**5A**). It was found that the percentage of MDSCs in patients who responded effectively to antibiotic treatment decreased. (**Figures 3B, D**, P<0.001), and the expression of immune checkpoint markers (Siglec-9, Tim-3 and PD-1) on MDSCs also decreased (**Figures 4B–G**, P<0.001). The percentage of MDSCs in patients who responded ineffectively to antibiotic treatment increased (**Figures 3C, E**, P<0.001), and the expression of the immune checkpoint marker Siglec-9 on MDSCs also increased (**Figures 5D, G**, P<0.001). However, the expressions of Tim-3 and PD-1 did not change significantly (**Figures 5B, C, E, F**, ns). Meanwhile, we discovered the serum concentrations Arg-1 and IL-6 of patients with ineffective antibiotic treatment also increased (**Figures 6A–D**, P<0.001,P<0.05).

### 3.5 Correlation between immune parameters

**Figure 7:**
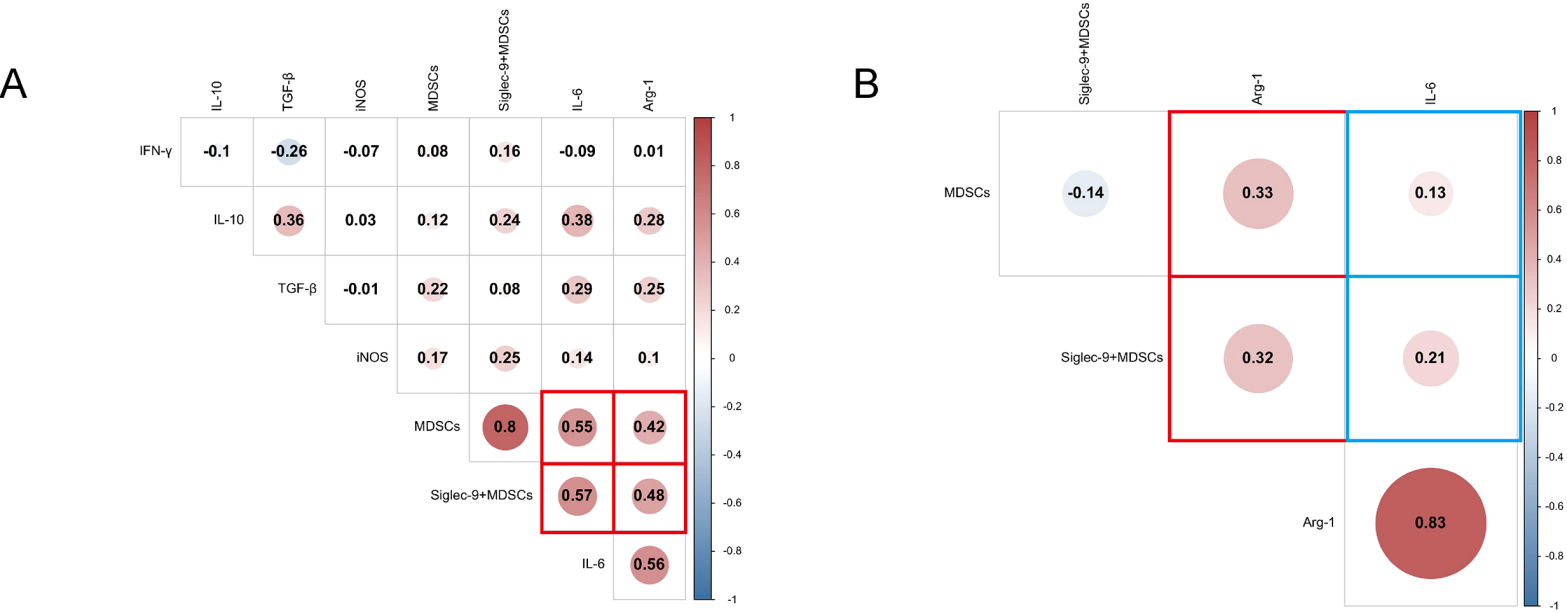
Correlation analysis heatmap. **(A)** Correlation analysis of cytokines and MDSCs, Siglec-9^+^ MDSCs in ABI and CBI patients. **(B)** Correlation analysis of Arg-1, IL-6 and MDSCs, Siglec-9^+^ MDSCs in patients with ineffective antibiotic treatment.

We conducted a correlation analysis of cytokines concentrations and the percentage of MDSCs and Siglec-9^+^MDSCs in ABI and CBI patients (**Figure 7A**), and found that Arg-1 and IL-6 were respectively correlated with MDSCs and Siglec-9^+^MDSCs. Furthermore, We conducted a correlation analysis of Arg-1, IL-6 and MDSCs, Siglec-9^+^MDSCs in patients with ineffective antibiotic treatment (**Figure 7B**). We observed no correlation between IL-6 and immune cell parameters. However, Arg-1 were correlated with MDSCs and Siglec-9^+^ MDSCs.

## 4 Discussion

MDSCs have been extensively studied in the context of cancer and autoimmune diseases. On one hand, in cancer, MDSCs support tumor growth and dissemination, suppress host immune responses, and evade immune surveillance. On the other hand, in autoimmune diseases, MDSCs often exhibit pro-inflammatory effects, exacerbating immune dysregulation (Shi et al., 2025). In recent years, MDSCs have been found to play a role in acute or chronic infectious diseases, such as infections caused by Mycobacterium tuberculosis, Staphylococcus aureus, and sepsis (Magcwebeba et al., 2019; Barrios et al., 2024). In our study, we discovered that Brucella infection can lead to an increase of MDSCs in patients, with a higher percentage of MDSCs observed in CBI patients compared to ABI patients. Research has commonly reported that Arg-1 and iNOS are two primary cytokines through which MDSCs exert immunosuppressive functions (Yamada et al., 2018; Wang et al., 2022). In our study, the concentrations of Arg-1 and iNOS in patients were found to be higher than those in HC subjects, with Arg-1 expression significantly elevated in CBI patients compared to ABI patients. This suggests that Arg-1 may play a role in long-term Brucella infection. A similar phenomenon was observed in the detection of IL-6. However, while the iNOS levels in patients were higher than those in HC subjects, no differences were found between ABI and CBI patients. Additionally, we measured the concentrations of IL-10, TGF-β, and IFN-γ. The results indicated that the levels of IL-10 and TGF-βin patients were higher than those HC subjects, yet no differences were observed between ABI and CBI patients. IFN-γlevels were highest during the acute phase and showed a decline in the chronic phase, suggesting that IFN-γmay be associated with acute infections.

Immune checkpoints markers primarily refer to inhibitory molecules expressed on immune cells that help regulate excessive immune responses under normal conditions. However, in the context of cancer or infectious diseases, these negative regulatory markers often exert immunosuppressive effects to evade immune surveillance, thereby facilitating tumor growth and bacterial survival (Li et al., 2021). Immune checkpoint markers such as PD-1 and Tim-3 have been deeply investigated in adaptive immune responses (Nakamura and Smyth, 2020). Hou et al. discovered that local irradiation can induce an increase in MDSCs systemically along with elevated expression of PD-1, while inhibition of PD-1 or MDSCs can eliminate radiotherapy-induced metastasis and improve clinical outcomes for patients (Hou et al., 2024b). In an acute sepsis model, PD-1 is highly expressed on MDSCs, and significantly inhibits T cell proliferation (Ruan et al., 2020). Dong et al. found that the expression of Tim-3 on MDSCs in patients with preeclampsia was higher than that in HC subjects, and blocking Tim-3 could weaken the suppressive function of MDSCs (Dong et al., 2021). Recent studies have found that MDSCs in the blood of lung cancer patients and tumor-bearing mice strongly express inhibitory Siglec receptors. Blocking Siglec receptors can significantly reduce the suppressive potential of MDSCs (Wieboldt et al., 2024). At present, antibiotic treatment is the main method for combating human brucellosis, including doxycycline and rifampicin (Bosilkovski et al., 2021). In this study, antibiotics were used to treat 37 ABI and 46 CBI. After the treatment, it was found that only 35 patients conditions were relieved, while 48 patients still had chronic symptoms. Flow cytometry detection revealed that the percentage of MDSCs, PD-1^+^MDSCs, Tim-3^+^MDSCs and Siglec-9^+^MDSCs of patients who responded effectively to antibiotic treatment decreased. In addition, the percentage of MDSCs and Siglec-9^+^MDSCs of patients who responded ineffectively to antibiotic treatment increased, but the expressions of PD-1^+^MDSCs and Tim-3^+^MDSCs showed no significant changes. Moreover, the detection of serum cytokines in patients with ineffective treatment revealed that the concentrations of Arg-1 and IL-6 were also elevated. Arg-1 has been proven to be one of the important cytokines determining the immunosuppressive function of MDSCs. Based on the ELISA results, we conducted a correlation test on ABI and CBI patients and found that there was a correlation between serum Arg-1, IL-6 and MDSCs, Siglec-9^+^ MDSCs. Therefore, we once again used ELISA to measure the concentrations of IL-6 and Arg-1 in the serum of patients with antibiotic ineffective treatment. Compared with before the treatment, the concentrations of IL-6 and Arg-1 both increased. Correlation tests conducted on patients with antibiotic ineffective treatment revealed that only Arg-1 was correlated with MDSCs and Siglec-9^+^ MDSCs. IL-6 did not show any correlation. Further support suggests that the immune checkpoint marker Siglec-9 may be associated with chronic infection and represents a potential prognostic marker for Brucella infection. However, further research is needed to confirm this specific phenomenon.

## Data Availability

The raw data supporting the conclusions of this article will be made available by the authors, without undue reservation.
